# Influence of Physical Education on Moderate-to-Vigorous Physical Activity of Urban Public School Children in St. Louis, Missouri, 2011–2014

**DOI:** 10.5888/pcd12.140458

**Published:** 2015-03-12

**Authors:** Susan B. Racette, Tiffany C. Dill, M. Leanne White, Jacqueline C. Castillo, Mary L. Uhrich, Cindi L. Inman, Nicholas C. DuPont, B. Ruth Clark

**Affiliations:** Author Affiliations: Tiffany C. Dill, Jacqueline C. Castillo, Mary Uhrich, Cindi L. Inman, Nicholas C. DuPont, B. Ruth Clark, Washington University School of Medicine, St. Louis, Missouri; M. Leanne White, St Louis Public Schools, St. Louis, Missouri.

## Abstract

We quantified the moderate-to-vigorous physical activity (MVPA, heart rate ≥140 bpm) of urban public elementary school children on school days with and school days without physical education (PE) class by using continuous heart rate monitoring. The heart rate of 81 students (93.8% black) in grades 3 and 5 was recorded in 15-second intervals. On the basis of 575 school-day observations (mean 7.1 days/student), students accumulated 44.4 (standard deviation [SD], 34.4) minutes of MVPA on days with PE and 30.6 (SD, 29.9) MVPA minutes on days without PE (*P* < .001). School policies should promote daily PE to help children in under-resourced areas achieve the recommended 60 minutes per day of MVPA.

## Objective

*Physical Activity Guidelines for Americans* (1) recommends that school-aged children engage in 60 minutes of physical activity daily, most of which should be moderate-to-vigorous physical activity (MVPA) because of its benefits to fitness, body composition, and metabolic health. However, children achieve on average only 30 MVPA minutes daily (2), and US children are less physically active than children in other countries (3). Urban children are particularly susceptible to inadequate physical activity (4). Physical education (PE) and recess provide MVPA opportunities in school, but policies vary widely (5). Currently, only 16 US states specify PE time requirements and only 9 states require recess in elementary schools. The aim of our study was to determine the contribution of PE class to the MVPA of urban public school children by using continuous heart rate monitoring.

## Methods

This observational, cross-sectional study was conducted during 3 school years (2011–2012, 2012–2013, and 2013–2014) at 4 urban public elementary schools in St. Louis, Missouri, with 95% student eligibility for the National School Lunch Program. Participants in grades 3 and 5 gave oral assent, and parents and guardians gave written consent. This study was approved by the Washington University School of Medicine Institutional Review Board and the school district’s Research Review Committee.

Heart rate was monitored continuously and recorded every 15 seconds using Polar E600 heart rate monitors (Polar Electro) during the school day for 1 to 4 school weeks from November through April. Each heart rate value was categorized as PE class, recess, lunch, or remainder of the school day and was based on the time associated with each heart rate value and each classroom’s daily schedule. PE classes were 50 minutes, once or twice each week. Recess occurred daily for 15 to 20 minutes, either outside (weather permitting) or inside (gymnasium or classroom). Each school day was coded as a PE day or non-PE day. Data on 81 students from the 4 schools and 3 school years were combined, and analyses were performed on the aggregate sample. All students had heart rate data for PE days and non-PE days.

Primary outcomes were MVPA minutes on days with PE class and days without PE and the proportion of time spent in MVPA during PE and recess. MVPA was defined as a heart rate at or above 140 beats per minute (bpm) (6); the proportion of time in MVPA was computed by dividing the number of 15-second heart rate values at or above 140 bpm by the total number of 15-second heart rate values during that segment of the school day. As a quality control measure, heart rate values lower than 50 bpm and greater than 215 bpm were excluded from analysis (7).

Additional outcomes included resting heart rate, estimated by averaging the 4 lowest consecutive heart rate values during classroom time each day; daily in-school steps quantified using Omron HJ–151 pedometers (Omron Healthcare, Inc) to complement the heart-rate data; body mass index (BMI) percentiles computed from measured weights and heights; and aerobic fitness estimated with the FITNESSGRAM 20-meter Progressive Aerobic Cardiovascular Endurance Run (8).

Generalized estimating equations with an exchangeable correlation matrix were used to evaluate the influence of PE class on daily in-school MVPA (determined by heart rate monitoring) and to compare the proportion of time in MVPA during PE and recess (SAS version 9.3, SAS Institute Inc). Pedometer step counts on days with and days without PE were compared by using analysis of variance.

## Results

Participants were 81 students (57% boys, 93.8% black) in grades 3 and 5 with a mean age of 10.5 years (SD, 1.0 y). Based on BMI, 11.3% of students were overweight and 17.5% were obese. Low aerobic fitness was observed among 26.3% of students, with a difference (*P* = .01) between the proportion of boys (15.6%) and girls (40.0%) who did not achieve the FITNESSGRAM Healthy Fitness Zone.

We obtained 575 days of heart rate observations (mean, 7.1 school days per student; SD, 3.5 days); 229 were school days with PE class, and 346 were school days without PE. Median heart rate values were 128 bpm during PE class, 115 bpm during recess, 109 bpm during lunch, and 101 bpm during the remainder of the school day. Resting heart rate was 77 bpm.

The average number of in-school MVPA minutes was greater on days with PE (44.4 min; SD, 34.4 min) than on days without PE (30.6 min; SD, 29.9 min, *P* < .001) (Figure). On average, students accumulated 17.1 MVPA minutes (SD, 9.9 min) during a 50-minute PE class (range, 0.0–40.8 min). Recess contributed an average 5.5 MVPA minutes (SD, 6.0 min) each school day, with no difference between days with and days without PE (*P* = .53). The proportion of time spent in MVPA was 38.3% (SD, 21.7 %) during PE class and 27.9% (SD, 28.0%) during recess (*P* = .001).

**Figure:**
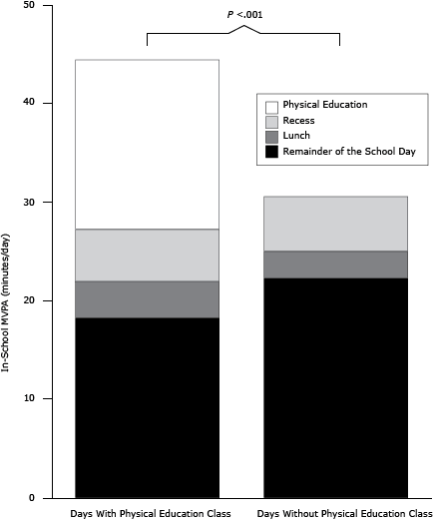
Moderate-to-vigorous physical activity (MVPA) of urban public school children in St. Louis, Missouri, 2011–2014, during several school days. Average minutes of in-school MVPA (defined as heart rate ≥140 bpm) were computed from 15-second heart rate values of 81 elementary school students. There were 229 observations on school days with physical education (PE) class and 346 observations on school days without PE class. The *P* value compares total minutes of MVPA on school days with PE class and school days without PE class. Abbreviations: MVPA, moderate-to-vigorous physical activity; PE, physical education. **Table:** 

In-School MVPA	Days With PE Class, min/d	Days Without PE Class, min/d
Recess	5.4	5.6
Lunch	3.6	2.8
Physical education class	17.1	Not applicable
Remainder of the school day	18.3	22.2

The influence of PE on MVPA minutes was similar for boys and girls (*P* = .82 for PE by sex interaction) and across schools (*P* = .65 for PE by school interaction), but was greater for 5th grade than for 3rd grade students (*P* = .04 for PE by grade interaction). Pedometer step counts indicated greater physical activity on days with PE class (4,437 steps/d; SD, 2,477 steps) than on days without PE class (3,628 steps/d; SD 1,695 steps, *P* < .001).

## Discussion

The major finding of this study was that PE class and recess provide opportunities for urban elementary school children to engage in MVPA during the school day. Racial/ethnic minority children residing in low-resource areas may benefit the most from school-based opportunities for MVPA. Our observation that less than 40% of PE class time was spent in MVPA is consistent with previous findings among 3rd grade students across 10 cities (9) and highlights the need for PE classes to be held more than once or twice each week.

Our results support recent recommendations (10) that the US Department of Education designate PE as a core curricular subject. Nationwide reductions in PE occurred as a consequence of the No Child Left Behind legislation (10). Importantly, both physical activity and physical fitness have been associated with greater academic achievement in several studies (11,12). A 2013 Cochrane Review (13) highlighted the benefits of school-based physical activity initiatives on increasing MVPA and fitness.

The White House Task Force on Childhood Obesity (14) identified “increasing physical activity” as one of 4 priority areas for enhancing child health. Notable recommendations are to increase the quality and frequency of PE and to promote recess for elementary school students. The Institute of Medicine (10) and an expert panel of the National Heart, Lung, and Blood Institute (15) recommended that elementary schools offer PE daily. Our results support these recommendations.

Limitations of this study are that heart rate was monitored during the school day only, and the sample was limited to 81 students in 4 schools. Strengths include the 15-second heart rate sampling periods as an index of physical activity intensity and the large number of sampling days.

In summary, physical education and recess provide essential opportunities for urban, racial/ethnic minority children to engage in physical activity of moderate or vigorous intensity during the school day. School policies should endorse daily PE and recess in elementary schools to facilitate achievement of MVPA goals.
